# Polycystic Ovary Syndrome May Be Associated With a Novel Mitochondrial tRNA^Asp^ Mutation

**DOI:** 10.1155/humu/6663471

**Published:** 2025-10-07

**Authors:** Yu Ding, Xuejiao Yu, Jian Xu, Caijuan Zhang, Jianhang Leng

**Affiliations:** ^1^Department of Laboratory Medicine, Hangzhou First People's Hospital, Hangzhou, China; ^2^Department of Laboratory Medicine, Quzhou People's Hospital, the Quzhou Affiliated Hospital of Wenzhou Medical University, Quzhou, China; ^3^Department of Gynecology and Obstetrics, Hangzhou First People's Hospital, Hangzhou, China

**Keywords:** mitochondrial dysfunction, mt-tRNA^Asp^, novel m.7544C>T mutation, PCOS-IR

## Abstract

Polycystic ovary syndrome is a common clinical condition often linked to insulin resistance (IR) and primarily affects women at reproductive age. Previous research has indicated a close association between mitochondrial tRNA (mt-tRNA) mutations and this syndrome; however, the range of mt-tRNA mutations in PCOS-IR remains largely unclear. In this study, we examined mt-tRNA mutations in 302 Han Chinese women with PCOS-IR and 589 control subjects, identifying a novel m.7544C>T mutation potentially related to this syndrome. At the molecular level, the m.7544C>T mutation occurs at a highly conserved nucleotide within the anticodon stem of mt-tRNA^Asp^, disrupting the 30C-40G base-pairing. Using cybrids cells derived from two individuals carrying this mutation and two controls without it, we observed that the m.7544C>T decreased the steady-state levels of tRNA^Asp^, altered mitochondrial RNA transcripts, impaired the activities of respiratory chain enzymes and oxygen consumption rates (OCRs), compromised mitochondrial functions, and increased oxidative stress. Overall, our findings strongly suggest that the m.7544C>T mutation contributes to the development of PCOS-IR, offering new insights into the pathophysiology of PCOS-IR driven by tRNA mutation–induced mitochondrial dysfunction and oxidative stress.

## 1. Introduction

Polycystic ovary syndrome (PCOS) is a prevalent endocrine disorder, affecting 5%–20% of women of reproductive age [1]. Besides its impact on fertility, PCOS is linked to an increased risk of obesity, IR, and metabolic syndrome (MetS) [2]. Growing evidence from in vitro and in vivo studies pointed to hyperandrogenemia and IR as key factors in the pathogenesis of this syndrome [3]. It has been suggested that 50%–70% of women with PCOS have IR at different levels [4], which impairs the responsiveness of muscle, liver, and adipose tissues to insulin and reduces glucose uptake. Mitochondria maintain cell energy status of oxidative phosphorylation (OXPHOS), which generates ATP essential for numerous cellular processes [5]. Consequently, mitochondrial dysfunction has often been proposed as a potential mechanism contributing to PCOS [6]. mtDNA is small, circular, double-strand, and contains 37 genes, among which 13 belong to components of respiratory chain, two ribosomal RNAs, and 22 transfer RNAs [7]. Because there are no histones to provide stability to mtDNA, combined with an inefficient DNA repair system in the cell nucleus and also absence of intracellular compartmentalization, the mutation rate of mtDNA could be higher than nuclear DNA (nDNA) by 100 folds [8]. Despite this, previous genome-wide association studies (GWAS) on PCOS have not systematically considered mtDNA mutations, suggesting that some of the unexplained heritability of the syndrome may stem from variations in the mitochondrial genome.

Previously, we screened for mtDNA mutations/variants in women with PCOS and found that most pathogenic mtDNA mutations were located in OXPHOS and mt-tRNA genes [9–12]. The functional analysis showed that, compared with the controls, PCOS-associated mt-tRNA mutations decreased the mtDNA copy number, ATP level, and the mitochondrial membrane potential (MMP) and increased the production of reactive oxygen species (ROS) and 8-hydroxy-2⁣′-deoxyguanosine (8-OhdG) [12]. Nevertheless, due to limited sample size, the type and association of the mutations in the 22 mt-tRNA genes in a large population with PCOS-IR are still unclear.

Here, we performed extended screening of the 22 mt-tRNA genes on 302 PCOS-IR patients and 589 controls, respectively. Through mutational analysis, 43 mutations/variants were identified across 21 distinct mt-tRNA genes. Notably, a novel mutation in tRNA^Asp^ (m.7544C>T) was detected in two genetically unrelated individuals with PCOS-IR. To better understand the functional impact of the m.7544C>T, we generated cytoplasmic hybrid (cybrid) cell lines with and without the m.7544C>T mutation in order to explore the underlying mechanisms contributing to PCOS-IR through determining the steady levels of mt-tRNA^Asp^, as well as mitochondrial functions within cybrids.

## 2. Materials and Methods

### 2.1. Study Population

From January 2013 to January 2022, 302 genetically unrelated women diagnosed with PCOS-IR and 589 age-matched healthy controls were recruited from Hangzhou First People's Hospital. Every subject received a questionnaire, personal medical history, and clinical inquiry, and the presence or absence of their health conditions was examined. Informed consent was signed by each participant. All samples were collected following an approach that was approved by the Ethics Committee of Hangzhou First People's Hospital (No. 2020-370-01); written informed consent was given by each subject involved in this investigation.

PCOS was diagnosed according to the revised 2003 Rotterdam criteria [13], requiring at least two of the following features: (1) oligoovulation/anovulation; (2) clinical or biochemical signs of hyperandrogenism; (3) polycystic ovarian morphology. Patients who have other diseases that can present symptoms similar to PCOS were excluded, such as non-classic adrenal hyperplasia, androgen-secreting tumors, and Cushing's syndrome.

Subjects with regular menstrual cycles, normal androgen levels, and no endocrine or systemic diseases, which could influence reproductive physiology and pregnancy, were included as controls.

### 2.2. Laboratory Assessments

Blood samples were drawn from all subjects between 7:00 AM and 10:00 AM after an overnight fast. Hormonal and biochemical examinations were carried out for all individuals enrolled in the present study. Body mass index (BMI) was calculated by dividing body weight (kg) with height's square (height × height) (m^2^). The luteinizing hormone (LH), follicle-stimulating hormone (FSH), prolactin (PRL), dehydroepiandrosterone sulfate (DHEA-S), total testosterone (TT), and fasting insulin (FINS) concentrations were evaluated using electrochemiluminescence immunoassays (Roche, Indianapolis, United States). Serum fasting plasma glucose (FPG), lactate, and creatine kinase (CK) concentrations were determined by routine methods (Beckman Coulter AU5800; Tokyo, Japan). Homeostasis model assessment of IR (HOMA-IR) test was used to assess IR in the case of hyperinsulinemia, HOMA-IR = (insulin × glucose)/22.5, and a score greater than or equal to 2.69 suggested IR [14].

### 2.3. Analysis of mt-tRNA Gene Mutations

Total genomic DNA was extracted from peripheral blood leukocytes of all subjects involved in this study by using the QIAamp Tissue Kit (Qiagen, Valencia, United States), in accordance with the manufacturer's instruction. PCR was carried out using 13 primers covering the whole mt-tRNA regions, based on the study as previously reported [15]. The PCR products were subsequently purified and sequenced by an ABI 3700 automatic DNA sequencer (Applied Biosystems, California, United States). In addition, the whole mtDNA genes of two probands (P1 and P2) who had the tRNA^Asp^ 7544C>T were amplified by 24 overlapping fragments [15]. The resulting sequence data of all mtDNA genes were aligned to the revised Cambridge Reference Sequence (rCRS, GenBank accession no. NC_012920.1) [16]. The mutations/variants in mtDNA were detected using software package “DNA STAR,” Version 5.01 (Madison).

### 2.4. Evaluations of the Pathogenic mt-tRNA Mutations

Fifteen species for the inter-specific comparison were selected, and the degree of conservation of a nucleotide was further evaluated by conservation index (CI), which represented the ratio of the species that shared the same nucleotide at a certain position in genome as that of *Homo sapiens*. CI ≥ 75% suggested functional importance [17].

The following criteria were used to screen the pathogenic/likely pathogenic mt-tRNA mutation: (1) presented in < 1% of the controls; (2) CI ≥ 75%; (3) potential structural and functional alterations; (4) impaired mitochondrial functions.

### 2.5. Generation of Cybrid Cell Models

The platelets were isolated from 3 mL of peripheral blood derived from two patients with the m.7544C>T mutation (P1 and P2), as well as two controls without this mutation (C1 and C2). The platelets were subsequently fused with 143B-derived mtDNA-less cells (*ρ*^0^ 206) [18]. The *ρ*^0^ 206 cells were cultured under high-glucose DMEM (Sigma-Aldrich, Darmstadt, Germany), which contained glucose (4.5 mg), pyruvate (0.11 mg/mL) and uridine (50 *μ*g/ml), suppled with BrdU (100 *μ*g/mL) and 10% FBS (Sigma-Aldrich, Darmstadt, Germany). The cybrid cell lines were routinely grown in the medium consisting of high-glucose DMEM with 10% FBS, 1% penicillin/streptomycin, and amphotericin B (0.25 *μ*g/mL).

We further used Next-Generation Sequence (NGS) to amplify the whole mitochondrial genomes using long-ranged PCR amplification from four cybrids. The NGS was carried out on the Illumina HiSeq 2000 sequencer (Illumina, California, United States), according to the manufacturer's guidelines [19]. Quality control was also performed on the original sequencing data, such as removing the low-quality sequence fragments, filtering out low-quality bases and low-coverage sequencing fragments. Moreover, Sanger sequencing was used to verify the presence of the m.7544C>T mutation. The forward primer sequences were 5⁣′-ACG CCA AAA TCC ATT TCA CT-3⁣′; reverse: 5⁣′-CGG GAA TTG CAT CTG TTT TT-3⁣′. The PCR products were purified and sequenced to detect the presence of m.7544C>T mutation (GenBank Accession No. NC_012920.1) [16].

### 2.6. Northern Blotting

To investigate the impact of the m.7544C>T mutation on tRNA metabolism, we conducted Northern blotting to assess the steady-state levels of tRNAs. Initially, total RNA was extracted from four cybrid cell lines using the TOTALLY RNA kit from Ambion (Thermo Fisher, Shanghai, China). An amount of 2 *μ*g of the extracted RNA was subjected to electrophoresis on a 10% polyacrylamide/7 M urea gel, followed by electroblotting onto a positively charged nylon membrane (Roche) for subsequent hybridization with specific oligodeoxynucleotide probes [20]. The sequences for digoxigenin (DIG)-labeled probes targeting tRNA^Asp^, tRNA^Leu(UUR)^, tRNA^Lys^, tRNA^Gln^, and 5S rRNA were as follows: 5⁣′-TAA GAT ATA TAG GAT TTA GCC TAT-3⁣′, 5⁣′-TGT TAA GAA GAG GAA TTG AAC CTC TGA CTG TAA-3⁣′, 5⁣′-TCA CTG TAA AGA GGT GTT GGT TCT CTT AAT CTT-3⁣′, 5⁣′-CTA GGA CTA TGA GAA TCG AAC CCA TCC CTG AGA-3⁣′, and 5⁣′-GGG TGG TAT GGC GGT AGAC-3⁣′.

### 2.7. Analysis of mt-RNA Transcription

The levels of mt-RNA for 13 OXPHOS subunits were then quantified using the 2^−*ΔΔ*Ct^ methodology [21]. Briefly, a total of 5 *μ*g of RNA was reverse-transcribed into cDNA with the Transcriptor First Strand cDNA Synthesis kit (Roche, Basel, Switzerland). Quantitative PCR (qPCR) was subsequently performed using the fluorogenic SYBR Green (Bio-Rad, California, United States), adhering to previously established protocols [21].

### 2.8. Analysis of Mitochondrial Respiratory Chain Complex Activities

Mitochondria were isolated from four cybrid cells on ice according to previously detailed methods [22], and the concentration of the mitochondrial fraction was evaluated using the BCA protein assay kit (Thermo Fisher Scientific, Madison, United States). The activities of Complexes I through IV were assessed and normalized against citrate synthase, following established descriptions [23].

### 2.9. Analyses of mtDNA Copy Number, ATP, MMP, NAD^+^/NADH Ratio, and Oxidative Stress–Related Biomarkers

Genomic DNA from two individuals with the m.7544C>T mutation and two control subjects were extracted from 3 mL of whole blood with the QIAamp Tissue Kit (Qiagen, Valencia, United States). The mtDNA copy number was quantified via real-time qPCR and 2^−*ΔΔ*Ct^ method, as described previously [24]. Cellular ATP content was measured with the Cell Titer-Glo Luminescent Cell Viability Assay (Promega, Madsion, United States) [25]. MMP was evaluated in four cybrid cell lines employing the JC-1 dye (Life Technology, California, United States) [26]. Additionally, the NAD^+^/NADH ratio was determined in cybrids using the WST-8 NAD^+^/NADH Assay kit (Beyotime, Shanghai, China), in accordance with the manufacturer's guidelines.

Moreover, the oxidative stress markers—including malondialdehyde (MDA), super oxide dismutase (SOD), glutathione peroxidase (GSH-Px), and ROS—were assessed in both control and mutant cell lines. MDA and SOD levels were evaluated using established methods from our previous study [27]. ROS was quantified with 2',7'-dichlorofluorescein diacetate (DCFH-DA) probe (Beyotime, Shanghai, China) [28]. Additionally, plasma 8-OhdG concentrations were determined via a competitive enzyme-linked immunosorbent assay (ELISA kit, Nikken Foods, Missouri, United States) [29]. All experiments were performed in triple.

### 2.10. Measurement of Oxygen Consumption Rate (OCR)

Mitochondrial respiration in four cybrid cell lines was evaluated with a Seahorse XF96 Extracellular Flux Analyzer (Seahorse Biosciences, Agilent, United States), following a previously described procedure [30]. Briefly, approximately 4 × 10^4^ cells per well were plated, and OCR was monitored using the Seahorse XF-24 system. Mitochondrial function was probed through the sequential injection of 1 *μ*M oligomycin, 1 *μ*M carbonyl cyanide-4-(trifluoromethoxy) phenylhydrazone (FCCP), and a mixture of 0.5 *μ*M rotenone with 1 *μ*M antimycin A [31]. All recorded OCR values were normalized to total protein content as determined by the Bradford assay, and subsequent data analysis was performed using the Seahorse Wave software.

### 2.11. Statistical Analysis

Statistical analysis was conducted with the SPSS software (Version 23.0). Continuous data were presented as mean ± standard deviation (SD) and were compared using the Student's *t*-test. For categorical variables, including the presence of mt-tRNA mutations in PCOS-IR and healthy control groups, the chi-square test was applied. A *p* value of less than 0.05 was considered statistically significant.

## 3. Results

### 3.1. Clinical Features

A total of 302 women diagnosed with PCOR-IR and 589 healthy control subjects were recruited from Hangzhou First People's Hospital. As summarized in Table 1, the PCOS-IR group exhibited significantly elevated levels of BMI, LH, LH/FSH ratio, DHEA-S, TT, FINS, CK, lactate, and HOMA-IR compared with the control group (*p* < 0.001). In contrast, no statistically significant differences were observed in FSH, PRL, or FPG between the two groups (*p* > 0.05).

### 3.2. Mutational Analysis of mt-tRNA Genes

In this study, mt-tRNA genes from all participants were fully sequenced. Identified mutations were cross-referenced with the MITOMAP, mtDB, and mtSNP databases. Alignment with the rCRS revealed 43 nucleotides across 21 mt-tRNA genes. Among these, eight mutations, specifically mt-tRNA^Leu(UUR)^ 3302A>G and 3275C>T, mt-tRNA^Gln^ 4395 T>C, mt-tRNA^Cys^ 5821C>G and 5802A>G, mt-tRNA^Ser(UCN)^ 7492C>T, mt-tRNA^Asp^ 7543A>G, and 7544C>T, disrupted Watson–Crick base pairs, suggesting potential structural and functional consequences for the mt-RNAs. Additionally, a novel mutation (mt-tRNA^Asp^ 7544C>T) was detected in two PCOS-IR patients but was absent in all 589 controls. This mutation was not documented in any of the referenced mitochondrial databases. Phylogenetic conservation analysis was conducted to determine the CI for each mutation. As shown in Table 2, the CI values varied widely, from 12.5% (mt-tRNA^Thr^ 15930G>A) to 100% (e.g., mt-tRNA^Cys^ 5802G>A, mt-tRNA^Asp^ 7544C>T, and mt-tRNA^Glu^ 14693A>G). Six mutations showed CIs exceeding 75%, 20 had CIs between 50% and 75%, and the rests were below 50%. Notably, 13 variants, including mt-tRNA^Phe^ 634 T>C, mt-tRNA^Ile^ 4277 T>C, mt-tRNA^Met^ 4454 T>C, mt-tRNA^Ser(UCN)^ 7498C>T, mt-tRNA^Gly^ 10007 T>C, and 10039A>G; mt-tRNA^His^ 12189 T>C, mt-tRNA^Glu^ 14727 T>C, mt-tRNA^Thr^ 15900 T>C, 15904C>T, 15930G>A, and 15938C>T; and mt-tRNA^Pro^ 15970 T>C, were exclusively present in the control group and absent in the PCOS-IR cohort, indicating they represented benign polymorphisms. Chi-square testing was applied to assess the distribution of mt-tRNA mutations between groups. Only the m.7544C>T mutation showed a statistically significant difference (*p* = 0.048), implying a potential association with PCOS-IR; no other mutations reached statistical significance.

**Figure 1:**
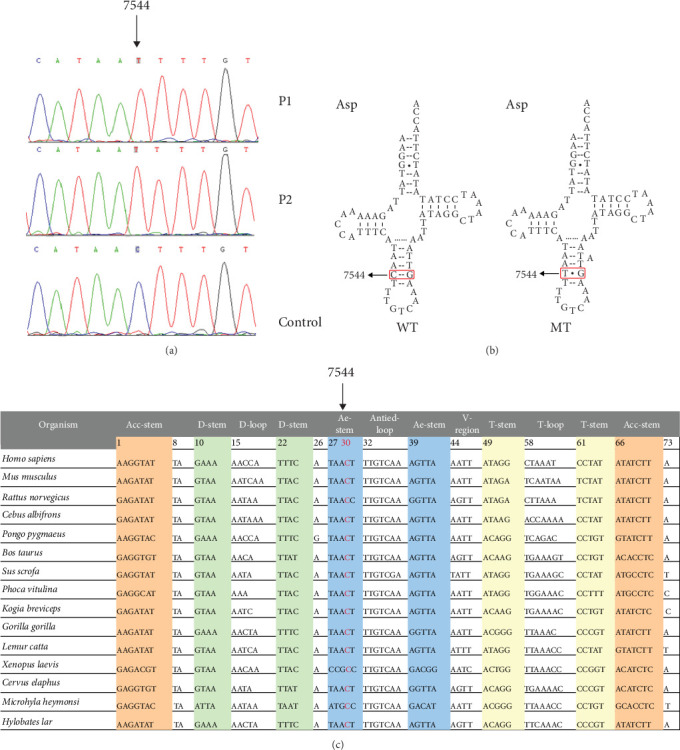
Molecular and genetic features of PCOS-IR associated 7544C>T mutation: (a) Identification of m.7544C>T mutation in two PCOS-IR patients (P1, P2). (b) The location of m.7544C>T mutation in mt-tRNA^Asp^, which disrupted 30C-40G base-pairing. (c) Alignment of mt-tRNA^Asp^ gene from different species; arrow indicates the Position 30, corresponding to the m.7544C>T mutation. MT, mutant; WT, wild type.

As depicted in Figure 1a,b, the homoplasmic m.7544C>T mutation was located at position 30 within the anticodon stem of mt-tRNA^Asp^. This mutation disrupted the conserved 30C-40G base-pairing, a pairing highly conserved across 15 species (Figure 1c). It is noteworthy that a mutation at an analogous site, m.12294G>A in mt-tRNA^Leu(CUN)^, had previously been associated with impaired mitochondrial function and linked to conditions such as pure exercise intolerance and ophthalmoplegia [32]. Therefore, we speculated that the m.7544C>T mutation may similarly cause mitochondrial dysfunction, particularly through destabilization of Watson–Crick pairing, thereby altering the structural and functional integrity of mt-tRNA^Asp^.

### 3.3. Clinical, Genetic, Molecular, and Biochemical Characteristics in Two PCOS-IR Carriers of the m.7544C>T Mutation

Two unrelated individuals with PCOS-IR were found to carry the m.7544C>T mutation. Detailed family and medical histories were collected to document any clinical symptoms. Both patients presented with Type 2 diabetes mellitus (T2DM), obesity, and PCOS-IR, but reported neither a family history of PCOS nor neuromuscular disorders. Additionally, Patient 1 was diagnosed with obstructive sleep apnea syndrome (OSAS), whereas Patient 2 showed moderate visual impairments (bilateral visual acuity: 0.1), and moderate hearing loss (right ear: 55 dB, left ear: 30 dB). Full clinical and laboratory profiles were provided in Table S1.

To assess the potential influence of mtDNA secondary variants on PCOS-IR, whole mitochondrial genomes sequencing was conducted in both carriers. Several genetic polymorphisms were detected (Table S2); however, phylogenetic conservation analysis indicated that only the m.7544C>T mutation was highly conserved, implying that other variants were unlikely to contribute significantly to PCOS-IR. These findings suggested that the m.7544C>T mutation was the primary factor underlying the clinical manifestations of PCOS-IR in these cases.

Further screening for the m.7544C>T mutation in the matrilineal relatives (mother and offspring) of both patients did not detect its presence, supporting the possibility that it may have arisen de novo.

### 3.4. Generation of Cybrid Cell Lines Carrying the m.7544C>T Mutation

To explore the pathogenicity of the m.7544C>T mutation, we generated cybrid cells carrying either the mutant or wild-type allele. Platelets isolated from two patients (P1 and P2), and two control individuals (C1 and C2) were fused with mtDNA-deficient *ρ*^0^ 206 cells. The resulting fusion products were cultured for 2 weeks in uridine- and sodium pyruvate-free medium to select for cybrid clones. The presence of the homoplasmic m.7544C>T mutation was confirmed by NGS, following a previously established method [33].

### 3.5. The m.7544C>T Mutation Reduced tRNA^Asp^ Abundance

**Figure 2:**
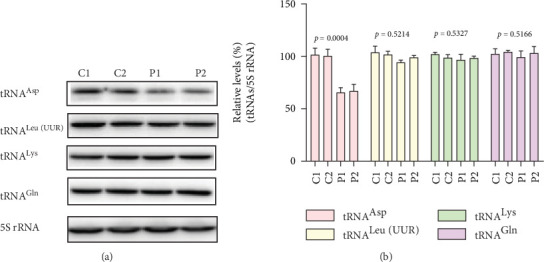
Northern blot analysis of tRNA: (a) Analysis of the steady-state levels of tRNA^Asp^, tRNA^Leu(UUR)^, tRNA^Lys^, tRNA^Gln^, and 5S rRNA in control and mutant cybrids. (b) Qualification of the relative levels of tRNA^Asp^, tRNA^Leu(UUR)^, tRNA^Lys^, and tRNA^Gln^.

To assess the impact of the m.7544C>T mutation on mt-tRNA metabolism, we performed Northern blot analysis of mt-RNAs from mutant and control cell lines using DIG-labeled probes specific for tRNA^Asp^, tRNA^Leu(UUR)^, tRNA^Lys^, and tRNA^Gln^. As illustrated in Figure 2a, the steady-state level of tRNA^Asp^ was markedly reduced in mutant cells, while the abundance of other tRNAs remained unchanged relative to controls. Quantitative analysis revealed a 38% decrease in tRNA^Asp^ levels following normalization to 5S rRNA (*p* = 0.0004; Figure 2b).

### 3.6. The m.7544C>T Mutation Impaired Mitochondrial Transcription

**Figure 3:**
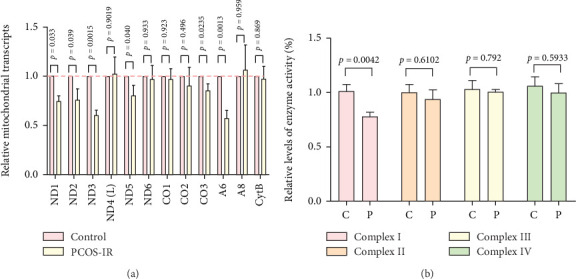
Analysis of OXPHOS functions in control and mutant cell lines. (a) Analyses of mt-RNA transcription in control and mutant cell lines. (b) Analysis of enzymatic activities of Complexes I–IV in control and mutant cell lines. C, controls; P, PCOS-IR.

We further analyzed mt-RNA transcription in cell lines derived from patients and controls. Significant reductions were observed in the mRNA levels of several mitochondrial genes—including ND1 (*p* = 0.033), ND2 (*p* = 0.039), ND3 (*p* = 0.0015), ND5 (*p* = 0.04), CO3 (*p* = 0.0235), and A6 (*p* = 0.0013)—in cells carrying the m.7544C>T mutation (Figure 3a). These findings indicated that the mutation may partially disrupt mt-RNA transcription.

### 3.7. Impaired Complex I Activity Caused by the m.7544C>T Mutation

To evaluate the impact of the m.7544C>T mutation on OXPHOS, we compared the enzymatic activities of respiratory chain complexes (I–IV) between mutant and control cell lines. As depicted in Figure 3b, Complex I activity was significantly reduced in mutant cells (*p* = 0.0042), whereas the activities of Complexes II, III, and IV remained unchanged (*p* > 0.05 for all).

### 3.8. Reduction in mtDNA Copy Number, ATP Level, MMP, and NAD^+^/NADH Ratio in Mutant Cell

**Figure 4:**
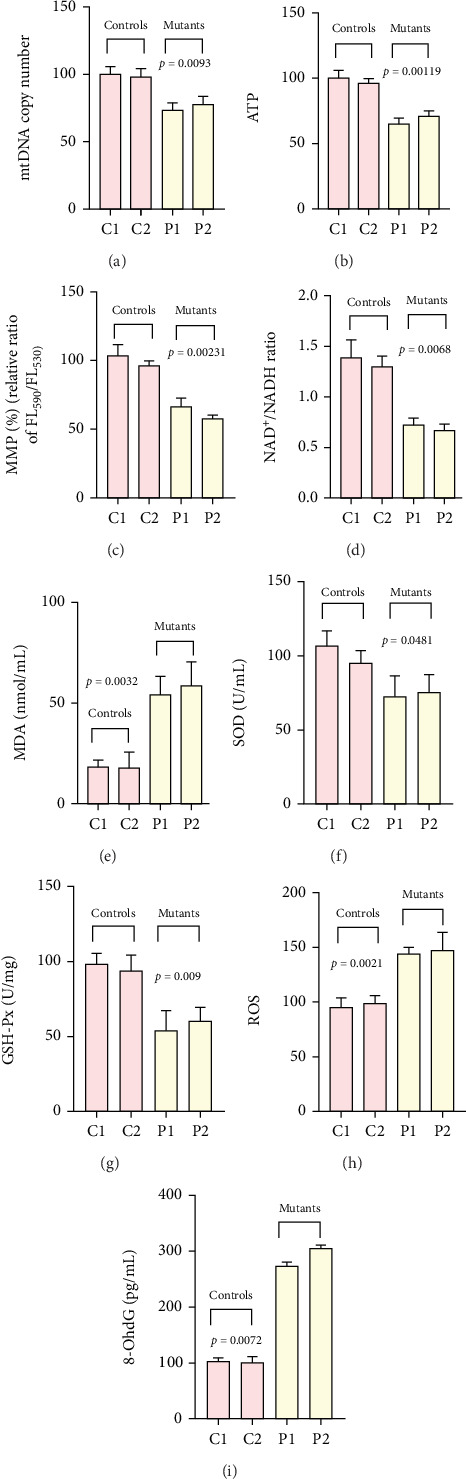
Determining the mitochondrial functions and oxidative stress–related biomarkers in control and mutant cell lines. (a) mtDNA copy number analysis; (b) ATP production; (c) MMP analysis; (d) NAD^+^/NADH ratio; (e) MDA analysis; (f) SOD analysis; (g) GSH-Px analysis; (h) ROS analysis; (i) 8-OhdG analysis.

Relative to control cybrids, cells harboring the m.7544C>T mutation exhibited decreased mtDNA copy number (*p* = 0.0093), ATP production (*p* = 0.00119), MMP (*p* = 0.00231), and NAD^+^/NADH ratio (*p* = 0.0068) (Figures 4a, 4b, 4c, and 4d). These results suggested a broad impairment of mitochondrial energy metabolism in mutant cells.

### 3.9. Elevated Oxidative Stress in m.7544C>T Carrying Cells

To assess whether the mutation disturbed redox homeostasis, we quantified the biomarkers related to oxidative stress. Mutant cell lines showed increased levels of MDA (*p* = 0.0032), ROS (*p* = 0.0021), and 8-OhdG (*p* = 0.0072), along with reduced activities of the antioxidant enzymes: SOD (*p* = 0.0481) and GSH-Px (*p* = 0.009) (Figures 4e, 4f, 4g, 4h, and 4i).

### 3.10. Altered OCR in Mutant Cybrids

**Figure 5:**
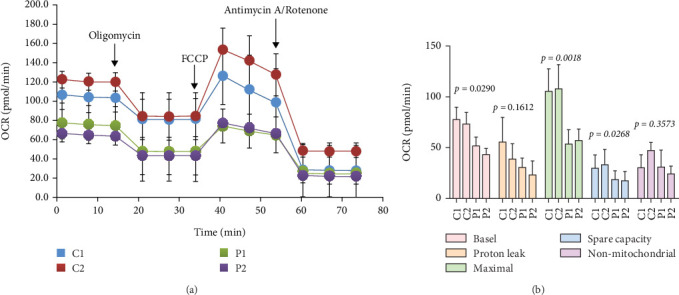
Respiration assays. (a) Analysis of OCR in control and mutant cell lines after treated with oligomycin, FCCP, antimycin A, and rotenone. (b) Qualification of the basal, proton leak, maximal, spare capacity, and non-mitochondrial OCRs in control and mutant cell lines.

We next investigated the bioenergetic profile of the cybrids by measuring OCR. Mutant cells demonstrated a significant reduction in basal (33.2% decrease, *p* = 0.029) compared with controls (Figure 5). Subsequent pharmacological profiling with oligomycin, FCCP, rotenone, and antimycin A revealed a pronounced decrease in maximal OCR (42.3% reduction, *p* = 0.0018). In contrast, proton leak, spare respiratory capacity, and non-mitochondrial oxygen consumption showed no statistical significance between the two groups (*p* > 0.05 for all).

## 4. Discussion

Due to its complex clinical presentation, the pathogenic mechanisms underlying PCOS remained poorly understood. As GWAS and other nuclear genomic investigations have provided limited mechanistic insight, research attention had shifted toward mitochondrial involvement [34]. Mitochondria played essential roles in cellular energy metabolism, apoptosis, and proliferation [11]. Within this organelle, mt-tRNAs acted as adapter molecules that translated genetic information into amino acid sequences, thereby supporting mitochondrial protein synthesis and respiratory chain function [35]. Although mt-tRNA genes represented only about 8% of the mitochondrial genome, the density of pathogenic mutations in these regions was notably high compared with that in mt-mRNAs, based on the relative lengths of their respective coding sequences [36]. Several case–control studies had recently attempted to link mt-tRNA mutations to PCOS-IR; for instance, the tRNA^Ser(AGY)^ 12267C>T and tRNA^Leu(CUN)^ 12308A>G variants were reported to correlate with PCOS-IR in a Pakistan cohort [37, 38]. However, these studies were constrained by small sample sizes and reliance on computational predictions, lacking functional validation to substantiate their claims.

To our knowledge, this represented the largest case–control genetic screening of mt-tRNA mutations in PCOS-IR. By applying stringent criteria—including evolutionary conservation, absent in controls, and in silico predictions of structural or functional impact, we identified a novel mt-tRNA^Asp^ 7544C>T mutation in two unrelated women with PCOS-IR. Structurally, this mutation occurred at a highly conserved site and disrupted a canonical Watson–Crick base pair (30C-40G). Using cybrid models, we demonstrated that the mutation reduced tRNA^Asp^ steady-state levels by approximately 38%, a decrease considered sufficient to elicit clinical phenotypes [39]. This tRNA deficiency likely contributed to reduce mRNA levels of ND1, ND2, ND3, ND5, CO3, and A6, and impaired Complex I activity—a critical and rate-limiting component of the OXPHOS system often implicated in mitochondrial diseases [40]. Impaired respiratory chain function was further reflected by reduced OCRs, underscoring the potential role of defective mt-tRNA metabolism in driving bioenergetic deficits.

The m.7544C>T mutation also induced broad mitochondrial dysfunctions, manifesting as significant reductions in mtDNA copy number, ATP levels, MMP, and NAD^+^/NADH ratio. mtDNA copy number served as an indicator of mitochondrial integrity and function [41], and its decline was linked to suppress transcription and diminished OXPHOS protein expression [42]. Altered MMP, which modulated apoptotic signaling and matrix organization [43], and a reduced NAD^+^/NADH ratio, which disrupted redox balance and energy metabolism [44], further illustrate the mutation's detrimental impact. Because insulin signaling relied on pathways such as PI3K/PKB and MAPK/ERK, mitochondrial dysfunction-induced interference with these cascades may contribute to IR and PCOS pathogenesis [45].

Mitochondria were central to redox homeostasis, and their dysfunction often elevated oxidative stress [46]. Consistent with this, we observed increased levels of MDA, ROS, and 8-OhdG, alongside decreased SOD and GSH-Px activities in mutant cells. Excessive reactive species can oxidize lipids, proteins, and nucleic acids, compromising cellular function and viability [47]. Given the importance of mitochondrial function in ovarian granulosa and theca cells, where they supported energy metabolism, steroidogenesis, and folliculogenesis [48, 49], the m.7544C>T mutation may disrupt ovarian physiology and contribute to anovulation, analogous to effects reported for the tRNA^Leu(UUR)^ 3302A>G mutation [17].

In summary, we identified a novel mt-tRNA^Asp^ 7544C>T mutation associated with PCOS-IR and demonstrated its detrimental effects on mitochondrial respiration and oxidative balance, providing new pathophysiological insights. Several limitations should be noted: Tissue-specific heteroplasmy was not assessed, mitochondrial haplogroup influences were not analyzed, and the sample size (302 patients) remained modest. Future studies involving larger cohorts and full mitochondrial genome sequencing were warranted to confirm these findings.

## Figures and Tables

**Table 1:** Clinical characteristics of women with PCOS-IR and control subjects.

**Characteristics**	**PCOS-IR (** **n** = 302**)**	**Control (** **n** = 589**)**	**p**
	**Mean ± SD (range)**	**Mean ± SD (range)**	
Age (y)	28.54 ± 4.79	26.14 ± 4.27	0.53
BMI (kg/m^2^)^a^	23.89 ± 3.21	19.12 ± 2.34	< 0.001
FSH (IU/L)	5.27 ± 1.38	6.79 ± 3.11	0.730
LH (IU/L)	11.44 ± 7.17	5.91 ± 3.71	< 0.001
LH/FSH ratio	2.12 ± 1.01	0.92 ± 0.69	< 0.001
PRL (*μ*g/L)	20.64 ± 7.85	13.19 ± 11.15	0.71
DHEA-S (*μ*mol/L)	9.22 ± 3.01	6.55 ± 2.61	< 0.001
TT (ng/mL)	0.75 ± 0.30	0.51 ± 0.17	< 0.001
FINS (mU/L)	16.74 ± 5.30	6.21 ± 1.55	< 0.001
FPG (mmol/L)	5.22 ± 1.51	5.19 ± 0.49	0.48
CK (U/L)	55.2 ± 3.55	22.5 ± 0.69	< 0.001
Lactate (mmol/L)	3.55 ± 0.88	1.01 ± 0.06	< 0.001
HOMA-IR^b^	3.28 ± 1.60	1.33 ± 0.20	< 0.001

Abbreviations: BMI, body mass index; CK, creatine kinase; DHEA-S, dehydroepiandrosterone sulfate; FINS, fasting insulin; FPG, fasting plasma glucose; FSH, follicle stimulation hormone; HOMA-IR, homeostasis model assessment of IR; LH, luteinizing hormone; PRL, prolactin; TT, total testosterone.

^a^BMI = weight (kg)/[height (m)]^2^.

^b^HOMA − IR = (fasting insulin) × (fasting glucose)/22.5.

**Table 2:** Mitochondrial tRNA variants in 302 women with PCOS-IR and 589 healthy controls.

**Gene**	**Mutation**	**CI (%)**	**Location of structure**	**Numbering of tRNA nucleotides** ^**a**^	**Watson–Crick base-pairing** ^**b**^	**PCOS-IR (%) (** **n** = 302**)**	**Controls (%) (** **n** = 589**)**	***p***	**Previously reported**
tRNA^Phe^	596T>C	43.9	D-arm	20		1 (0.33)	0 (0)	0.16	Yes
	634T>C	17.1	T*ψ*C loop	58		0 (0)	1 (0.17)	0.47	Yes
tRNA^Val^	1658T>C	51.2	T*ψ*C loop	61		1 (0.33)	0 (0)	0.16	Yes
tRNA^Leu(UUR)^	3302A>G	96.1	Acceptor arm	71	T-A↓	1 (0.33)	0 (0)	0.16	Yes
	3275C>T	86.6	Variable region	44	A-T↑	1 (0.33)	0 (0)	0.16	Yes
	3290T>C	31.2	T*ψ*C loop	59		3 (1.0)	3 (0.51)	0.40	Yes
tRNA^Ile^	4277T>C	21.2	D-arm	15		0 (0)	1 (0.17)	0.47	Yes
	4312C>T	19.5	T*ψ*C loop	54		1 (0.33)	0 (0)	0.16	Yes
tRNA^Met^	4454T>C	60.0	T*ψ*C loop	58		0 (0)	3 (0.51)	0.21	Yes
tRNA^Gln^	4363T>C	75.0	Anticodon stem	38		1 (0.33)	0 (0)	0.16	Yes
	4395T>C	57.1	Acceptor arm	6	C-G↑	1 (0.33)	0 (0)	0.16	Yes
tRNA^Trp^	5531A>G	24.4	D-arm	20		1 (0.33)	1 (0.17)	0.63	Yes
	5567T>C	68.3	T*ψ*C loop	61		1 (0.33)	1 (0.17)	0.63	Yes
tRNA^Ala^	5601C>T	63.4	T*ψ*C loop	59		2 (0.66)	3 (0.51)	0.77	Yes
	5603G>A	70	T*ψ*C loop	61		1 (0.33)	3 (0.51)	0.70	Yes
tRNA^Asn^	5711A>G	65.6	D-arm	19		1 (0.33)	0 (0)	0.16	Yes
tRNA^Cys^	5802G>A	100	Anticodon stem	30	G-C↓	1 (0.33)	0 (0)	0.16	Yes
	5811T>C	67.0	D-arm	16		1 (0.33)	2 (0.34)	0.98	Yes
	5821G>A	66.6	Acceptor arm	6	G-C↓	3 (0.99)	1 (0.17)	0.08	Yes
tRNA^Ser(UCN)^	7492C>T	73.1	Anticodon stem	26	A-T↑	1 (0.33)	0 (0)	0.16	Yes
	7498C>T	33.0	D-arm	17		0 (0)	2 (0.34)	0.31	Yes
tRNA^Asp^	7543A>G	73.3	Anticodon stem	29	A-T↓	1 (0.33)	0 (0)	0.16	Yes
	7544C>T	100	Anticodon stem	30	C-G↓	2 (0.66)	0 (0)	0.048	No
tRNA^Lys^	8343A>G	46.1	T*ψ*C loop	54		4 (1.32)	2 (0.34)	0.09	Yes
tRNA^Gly^	10007T>C	43.8	D-arm	20		0	1 (0.17)	0.47	Yes
	10031T>C	51.2	Variable region	44		3 (1.0)	2 (0.34)	0.98	Yes
	10039A>G	58.5	T*ψ*C loop	53		0 (0)	2 (0.34)	0.31	Yes
tRNA^Arg^	10454T>C	69.2	T*ψ*C loop	55		1 (0.33)	3 (0.51)	0.71	Yes
tRNA^His^	12153C>T	59.0	D-arm	16		1 (0.33)	1 (0.17)	0.63	Yes
	12188T>C	63.4	T*ψ*C loop	55		1 (0.33)	0 (0)	0.16	Yes
	12189T>C	36.5	T*ψ*C loop	56		0 (0)	4 (0.68)	0.15	Yes
tRNA^Ser(AGY)^	12234A>G	70.7	Acceptor arm	42		2 (0.66)	2 (0.34)	0.49	Yes
	12246C>A	50.0	T*ψ*C loop	54		1 (0.33)	0 (0)	0.16	Yes
tRNA^Leu(CUN)^	12280A>G	58.5	D-arm	15		1 (0.33)	1 (0.17)	0.63	Yes
tRNA^Glu^	14693A>G	100	T*ψ*C loop	54		3 (0.99)	3 (0.51)	0.40	Yes
	14727T>C	43.9	D-arm	18		0 (0)	1 (0.17)	0.47	Yes
tRNA^Thr^	15900T>C	46.6	D-arm	13		0(0)	1 (0.17)	0.47	Yes
	15904C>T	67.3	D-arm	17		0 (0)	2 (0.34)	0.31	Yes
	15907A>G	63.4	D-arm	22		1 (0.33)	0 (0)	0.16	Yes
	15930G>A	12.5	Variable region	45		0 (0)	3 (0.51)	0.21	Yes
	15938C>T	41.4	T*ψ*C loop	54		0 (0)	1 (0.17)	0.47	Yes
tRNA^Pro^	15970T>C	24.4	T*ψ*C loop	59		0 (0)	2 (0.34)	0.31	Yes
	16017A>G	35.0	Acceptor arm	7		1 (0.33)	1 (0.17)	0.63	Yes

Abbreviation: CI: conservation index.

^a^Numbers represent the nucleotide positions according to the mitotRNAdb (https://mttrna.bioinf.uni-leipzig.de/mtDataOutput).

^b^Watson–Crick base-pairing: created: ↑; disrupted: ↓.

## Data Availability

The datasets used and/or analyzed during the current study are available from the corresponding author on reasonable request (dingyu_zj@126.com).

## Supporting Information

Supporting Information 1 Table S1: Clinical and biochemical characteristics of two patients with the novel m.7544C>T mutation.

Supporting Information 2 Table S2: Analysis of mtDNA variants in two patients with PCOS-IR.
