# Supplementary material for: Polycystic Ovary Syndrome May Be Associated With a Novel Mitochondrial tRNAAsp Mutation
Source: Hum Mutat. 2025 Oct 7;2025:6663471. doi: 10.1155/humu/6663471 (PMC12520804; doi:10.1155/humu/6663471)
Supplement: Supporting Information 1 — Table S1: Clinical and biochemical characteristics of two patients with the novel m.7544C>T mutation. [file 6663471.f1.docx]

**Supplementary Table S1. Clinical and biochemical characteristics of two patients with the novel m.7544C>T mutation**

| Patients | Age  (Y) | BMI  (kg/m^2^) | HbA1c  (%) | Glucose (0h) (mmol/L) | Glucose  (2h)  (mmol/L) | FINS (mIU/L) | HOMA-IR | FSH (IU/L) | LH (IU/L) | LH/FSH ratio | PRL (μg/L) | TT  (ng/mL) | Visual  Acuity  Right/  Left  Eye | PTA  (dB)  Right/  Left  Ear | Family history |
| --- | --- | --- | --- | --- | --- | --- | --- | --- | --- | --- | --- | --- | --- | --- | --- |
| P1 | 28 | 25.4 | 6.9 | 7.3 | 13.5 | 9.2 | 2.98 | 4.8 | 8.4 | 1.75 | 14.6 | 1.02 | 0.4/0.3 | 20/25 | No |
| P2 | 32 | 26.0 | 7.1 | 8.0 | 14.7 | 10.6 | 3.77 | 6.3 | 10.1 | 1.60 | 13.9 | 0.89 | 0.1/0.1 | 55/30 | No |

BMI: body mass index; FINS: fasting insulin; HOMA-IR: homeostasis model assessment of insulin resistance; FSH: follicle stimulation hormone; LH: luteinizing hormone; PRL: prolactin; TT: total testosterone, PTA: pure-tone audiometry, dB, decibel
