# Supplementary material for: Polycystic Ovary Syndrome May Be Associated With a Novel Mitochondrial tRNAAsp Mutation
Source: Hum Mutat. 2025 Oct 7;2025:6663471. doi: 10.1155/humu/6663471 (PMC12520804; doi:10.1155/humu/6663471)
Supplement: Supporting Information 2 — Table S2: Analysis of mtDNA variants in two patients with PCOS-IR. [file 6663471.f2.docx]

**Supplementary Table S2. Analysis of mtDNA variants in two patients with PCOS-IR**

| **Gene** | **Position** | **Alterations** | **Conservation**  **(H/B/M/X)^a^** | **rCRS^b^** | **P1** | **P2** |
| --- | --- | --- | --- | --- | --- | --- |
| **D-loop** | 73 | A→G |  | A | G | G |
|  | 153 | A→G |  | A |  | G |
|  | 195 | T→C |  | T | C | C |
|  | 263 | A→G |  | A | G |  |
|  | 289 | T→C |  | T |  | C |
|  | 310 | T→CTC |  | T |  | CTC |
|  | 489 | T→C |  | T | C | C |
|  | 613 | A→G |  | A | G |  |
|  | 16051 | A→G |  | A |  | G |
|  | 16129 | G→A |  | G |  | A |
|  | 16189 | T→C |  | T | C |  |
|  | 16223 | C→T |  | C | T |  |
|  | 16274 | G→A |  | G | A | A |
|  | 16362 | T→C |  | T | C | C |
| **12S rRNA** | 709 | G→A | G/A/A/- | G |  | A |
|  | 750 | A→G | A/A/G/- | A |  | G |
|  | 1438 | A→G | A/A/A/G | A | G | G |
| **16S rRNA** | 2706 | A→G | A/G/A/A | A | G |  |
|  | 3010 | G→A | G/G/A/A | G | A | A |
|  | 3107 | Del N |  | N | Del N | Del N |
| ***ND1*** | 3483 | G→A |  | G |  | A |
|  | 3970 | C→T |  | C |  | T |
| ***ND2*** | 4769 | A→G |  | A | G | G |
|  | 4883 | C→T |  | C | T |  |
|  | 5230 | G→A |  | G | A |  |
|  | 5459 | T→C |  | T | C | C |
| ***COI*** | 5978 | A→G |  | A |  | G |
|  | 7028 | C→T |  | C | T | T |
| **tRNA^Asp^** | 7544 | C→T | C/C/C/C | C | T | T |
| ***COII*** | 7976 | G→A (Gly→Ser) | G/G/S/G | G | A |  |
| ***A8*** | 8414 | C→T (Leu→Phe) | L/F/M/W | C | T | T |
| ***A6*** | 8701 | A→G (Thr→Ala) | T/S/L/Q | A | G |  |
|  | 8860 | A→G (Thr→Ala) | T/A/A/T | A |  | G |
| ***COIII*** | 9540 | T→C |  | T | C | C |
|  | 9950 | T→C |  | T |  | C |
| ***ND3*** | 10398 | A→G (Thr→Ala) | T/T/T/A | T | A | A |
|  | 10400 | C→T |  | C | T | T |
| ***ND4*** | 10873 | T→C |  | T | C |  |
|  | 11059 | C→T |  | C |  | T |
|  | 11719 | G→A |  | G | A | A |
| ***ND5*** | 12705 | C→T |  | C | T | T |
|  | 12825 | T→C |  | T |  | C |
| ***ND6*** | 14668 | C→T |  | C | T |  |
| ***CytB*** | 14766 | C→T (Thr→Ile) | T/S/I/S | C | T | T |
|  | 14783 | T→C |  | T | C | C |
|  | 15043 | G→A |  | G | A | A |
|  | 15301 | G→A |  | G |  | A |
|  | 15326 | A→G (Thr→Ala) | T/M/I/I | A | G | G |
|  | 15784 | T→C |  | T |  | C |

^a^Conservation assessments in human (H), Bovine (B), Mouse (M) and *Xenopus laevis* (X).

^b^rCRS: reversed Cambridge Reference Sequences
